# MicroRNAs miR-148a-3p, miR-425-3p, and miR-20a-5p in Patients with IgA Nephropathy

**DOI:** 10.3390/genes16020125

**Published:** 2025-01-23

**Authors:** Jarosław Przybyciński, Michał Czerewaty, Ewa Kwiatkowska, Violetta Dziedziejko, Krzysztof Safranow, Leszek Domański, Andrzej Pawlik

**Affiliations:** 1 Department of Nephrology, Transplantology and Internal Medicine, Pomeranian Medical University, 70-111 Szczecin, Poland; jaroslaw.przybycinski@pum.edu.pl (J.P.); ewakwiat@gmail.com (E.K.); domanle@pum.edu.pl (L.D.); 2 Department of Physiology, Pomeranian Medical University, 70-111 Szczecin, Poland; michal.czerewaty@wp.pl; 3 Department of Biochemistry and Medical Chemistry, Pomeranian Medical University, 70-111 Szczecin, Poland; viola@pum.edu.pl (V.D.); chrissaf@mp.pl (K.S.)

**Keywords:** microRNAs, IgA nephropathy, kidney

## Abstract

Background/Objectives: IgA nephropathy (IgAN) is one of the most common forms of glomerulonephritis leading to renal failure. MicroRNAs have been shown to play an important role in the pathogenesis and clinical course of IgA nephropathy; therefore, they offer the possibility of noninvasive diagnosis of this disease and have some value in predicting disease prognosis. This study aimed to evaluate the plasma levels of miR-148a-3p, miR-425-3p, and miR-20a-5p in patients with IgA nephropathy and their correlation with selected clinical parameters. Methods: This study included 44 patients with IgA nephropathy and 46 control subjects. Results: The results of our study indicated that in patients with IgA nephropathy, the increased plasma levels of miR-148a-3p and miR-425-3p correlated negatively with eGFR values. According to the Haas classification, plasma levels of miR-20a-5p were statistically significantly increased in patients with histopathological changes classified as Stages 3, 4, and 5 compared with patients with histopathological changes classified as Stages 1 and 2. Conclusions: The results of our study suggest the possible involvement of miR-148a-3p, miR-425-3p, and miR-20a-5p in the pathogenesis of IgA nephropathy.

## 1. Introduction

IgA nephropathy (IgAN) is the most common primary proliferative glomerulonephritis, leading to end-stage renal disease (ESRD) in 20% to 40% of patients. Abnormal IgA1 immunoglobulin and immune complexes are involved in the pathogenesis of this disease [1]. Immunoglobulin A (IgA) exists in the form of two subtypes: IgA1 and IgA2. IgA1, in its monomeric form, is mainly present in the blood, while the dimeric form IgA2 is found primarily on the surface of mucous membranes. IgA1 and IgA2 have a different structure and function [2]. Differences in the number and location of the attached carbohydrate residues to the amino acid molecules affect the difference in the way the immunoglobulin binds to the receptor and triggers the immune response [3]. In patients with IgA nephropathy, immunoglobulin A1 does not contain galactose-deficient IgA1 (Gd-IgA1) molecules attached during glycosylation. A defect in IgA1 glycosylation results in the release of nascent IgA1 into the circulation in response to antigens on the surface of mucous membranes [4]. Its presence in the blood induces the formation of autoantibodies that combine with IgA1 to form immune complexes. The resulting immune complexes induce an inflammatory process. As abnormal IgA1 is formed after contact with antigens on the mucosal surface, there is increasing evidence linking the occurrence of IgAN to mucosal infections [5].

Under physiological conditions, in response to bacterial antigens, IgA2 is synthesized, whose main function is to neutralize toxins present on the surface of mucous membranes and prevent the penetration of pathogens through the mucosal barrier. The IgA1 formed in serum induces an inflammatory response by binding to receptors on the surface of monocytes, neutrophils, and some dendritic cells. In the development of IgAN, immune complexes with structurally altered IgA1 immunoglobulin are involved [6]. The presence of structurally altered immunoglobulin A1 (Gd-IgA1) induces the formation of autoantibodies. The Gd-IgA1-IgG immune complexes thus formed are deposited within the mesangium. In addition, Gd-IgA1 deposits can be produced in the mesangium, and the autoantibodies that bind to them form immune complexes [7]. Deposited immune complexes Gd-IgA1-IgG within the mesangium cells cause their proliferation, deposition of extracellular matrix, and increased synthesis of pro-inflammatory cytokines, such as interleukin-6 (IL-6), tumor necrosis factor α (TNFα), and transforming growth factor β (TGFβ), as well as activation of the complement system [8]. The induced inflammation causes damage to podocytes and the entire filtration barrier. Histopathologically, IgA nephropathy is classified as mesangial proliferative glomerulonephritis. The diagnosis of IgAN requires a renal biopsy [9]. The defining pathological feature is the presence of IgA in immune deposits in mesangial areas of the glomeruli. Glomerular IgA belongs to the IgA1 subclass. Other components that can be detected by immunofluorescence microscopy are the complement components C3 and C4, properdin, terminal complement complex (C5b-C9), and mannose-binding lectin [10]. This confirms the involvement of alternative and lectin pathways of complement activation in the pathogenesis of IgAN. The main symptoms of IgA nephropathy are hematuria and proteinuria. Usually, the amount of protein in the urine does not exceed 3 g/24 h. The diagnostic tests remain assessment of daily urinary protein excretion, quantitative assessment of albumin excreted in urine relative to creatinine excretion in the form of the albumin to creatinine ratio (ACR), or assessment of protein excreted in urine relative to creatinine excretion in the form of the protein to creatinine ratio (PCR) [11]. In clinical practice, urine microalbumin, 24 h urine protein, serum creatinine, and the glomerular filtration rate (GFR) are commonly used to assess the status and prognosis of IgAN. However, these indices have many influencing factors, and their sensitivity and specificity are poor. At present, new markers are still being sought that can help diagnose this disease and monitor and predict its course. In recent years, there has been increased interest in microRNA as potential markers of IgA neuropathy [12]. It was indicated that some microRNAs play an important role in the pathogenesis of the inflammatory response and renal fibrosis and influence the disease process’s development. MicroRNAs (miRNAs) are a class of non-coding RNAs that, although they do not have the ability to encode proteins, are able to influence the expression of many genes at the post-transcriptional level [13]. The action of miRNAs is to bind to response elements in the 3′-untranslated region (3′-UTR), leading to inhibition of mRNA translation. More than 60% of human protein-coding genes have been shown to be regulated by miRNAs. Since miRNAs are involved in modulating many genes’ expression, dysregulation of some miRNAs can cause or contribute to a wide variety of diseases in humans [14].

In addition to modulating gene expression and thus influencing the synthesis of many proteins, miRNAs are also used in clinical practice as noninvasive prognostic and predictive diagnostic biomarkers for many diseases. Previous studies have also shown that miRNAs play an important role in the development and progression of various glomerular diseases, indicating that they may also represent potential biomarkers and therapeutic targets for the diagnosis of and treatment of IgA nephropathy [15,16,17].

MicroRNAs that could potentially be involved in the pathogenesis of IgA nephropathy are miR-148a-3p, miR-425-3p, and miR-20a-5p. So far, only one study in a Chinese population has confirmed the possible association of these microRNAs with IgA nephropathy [18]. To date, many studies have been published on the role of miR-148a-3p, miR-425-3p, and miR-20a-5p in the pathogenesis of various diseases, including kidney diseases. Most studies have been performed on animal models or cell cultures. Previous studies have shown that these microRNAs are involved in a number of cellular processes and signaling pathways that may be implicated in the pathogenesis of IgA nephropathy. These microRNAs may influence fibrosis processes and endothelial function, affect immune cells and the immune response, and may be involved in renal ischemia-reperfusion injury [19,20,21,22,23].

The aim of this study was to evaluate the plasma levels of miR-148a-3p, miR-425-3p, and miR-20a-5p in patients with IgA nephropathy and their correlation with selected clinical parameters.

## 2. Materials and Methods

This study included 44 patients (23 male, 21 female) diagnosed with IgA nephropathy confirmed by renal biopsy, clinical symptoms, and biochemical parameters. The patients were treated at the Department of Nephrology at Pomeranian Medical University in Szczecin, Poland. On the basis of the renal biopsy, the patients were classified into five subgroups according to the Haas classification [24]. Histopathological preparations were also evaluated for the presence of focal segmental glomerular sclerosis. Baseline biochemical parameters were determined in all patients. Clinical data, including serum creatinine concentration and 24 h urinary protein excretion (UPE), were collected for all patients. A formula developed by the Chronic Kidney Disease Epidemiology Collaboration (CKD-EPI) was used to calculate the glomerular filtration rate. The control group consisted of 46 healthy subjects (27 male, 19 female). This study was conducted according to the guidelines laid down in the Declaration of Helsinki, and all procedures involving human subjects/patients were approved by the Ethical Committee of the Pomeranian University in Szczecin (Poland), number KB-0012/69/17.

### 2.1. miRNA Isolation

All 90 EDTA blood samples were centrifuged at 3000 rpm for 10 min. The whole plasma was collected in 1.5 mL collection tubes and immediately stored at −80 °C until the time of genetic analysis. The MagMAX™ mirVana™ Total RNA Isolation Kit (Applied Biosystems, Waltham, MA, USA) was used to extract total RNA (including microRNAs), following the manufacturer’s protocol. In addition, 5′-phosphorilated *Arabidopsis thaliana* ath-miR159a (5′-UUUGGAUUGAAGGGAGCUCUA-3′) (Thermo Fisher Scientific, Waltham, MA, USA) was added to each sample to monitor the efficiency of miRNA isolation according to the manufacturer’s recommendations. In the next step, cDNA was synthesized using the TaqMan™ Advanced miRNA cDNA Synthesis Kit (Applied Biosystems, Waltham, MA, USA) according to the manufacturer’s protocol.

### 2.2. Real-Time Quantitative Reverse Transcription PCR (RQ-PCR)

qRT-PCR reactions were performed on an ABI PRISM^®^ Fast 7500 Sequence Detection System (Applied Biosystems, Waltham, MA, USA) with TaqMan™ Fast Advanced Master Mix (Applied Biosystems, Waltham, MA, USA). The real-time conditions were as follows: 95 °C for 20 s, followed by 40 cycles at 95 °C for 3 s, and 60 °C for 30 s.

Many strategies have shown how to normalize the results for circulating miRNAs. None of these methods is fully acceptable and universal. hsa-miR191-5p was previously reported as an endogenous control for serum/plasma-circulating miRNA in various diseases [25,26,27,28,29,30,31,32].

Considering that hsa-miR-191 was also reported as a housekeeping miR to normalize urine-circulating miRNAs [33] and tissue miRNAs in patients with IgAN [34], according to the biotechnology company’s recommendations [35], we selected hsa-miR-191-5p as a normalizer.

All reactions were performed for selected miRNAs using TaqMan™ Advanced miRNA Assays (Applied Biosystems, Waltham, MA, USA) (Table 1).

### 2.3. Statistical Analysis

Plasma concentrations of miR-148a-3p, miR-425-3p, and miR-20a-5p were compared using the Mann–Whitney U test. Spearman’s rank correlation coefficients (R_s_) were used to measure the correlation between miR-148a-3p, miR-425-3p, and miR-20a-5p plasma concentrations and clinical parameters. A *p*-value < 0.05 was considered to be statistically significant.

## 3. Results

**Figure 1:**
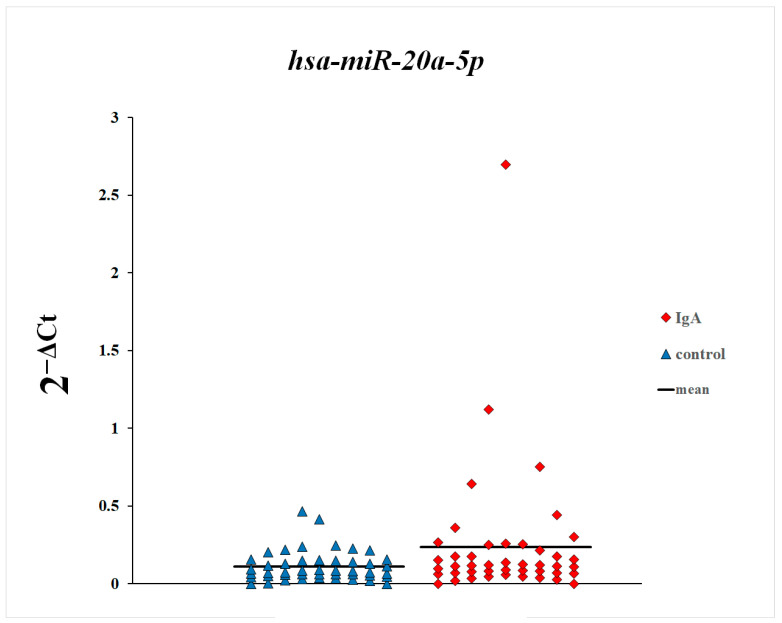
Plasma levels of miR-20a-5p in patients with IgA nephropathy and control subjects.

**Figure 2:**
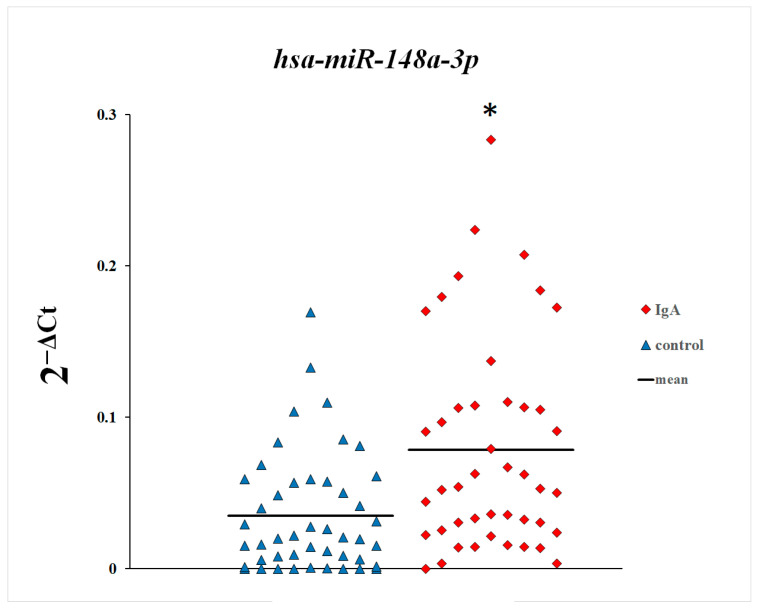
Plasma levels of miR-148a-3p in patients with IgA nephropathy and control subjects. * *p* = 0.0002, Mann–Whitney U test.

**Figure 3:**
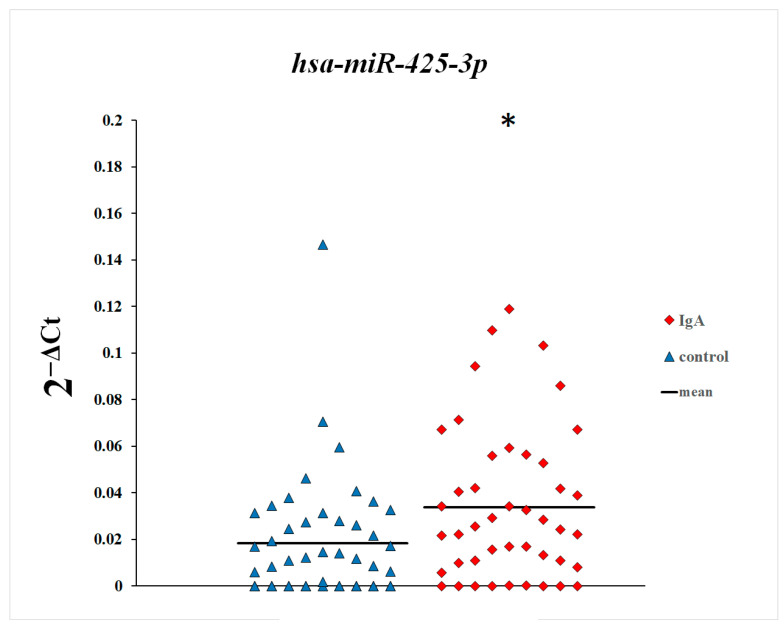
Plasma levels of miR-425-3p in patients with IgA nephropathy and control subjects. * *p* = 0.01, Mann–Whitney U test.

In the first step of this study, we compared plasma levels of microRNAs between patients with IgA nephropathy and control subjects. As shown in Figure 1, Figure 2 and Figure 3, plasma levels of miR-148a-3p and miR-425-3p in patients with IgA nephropathy were statistically significantly increased in comparison with the control group. There were no statistically significant differences in the plasma levels of miR-20a-5p between patients with IgA nephropathy and control subjects.

**Figure 4:**
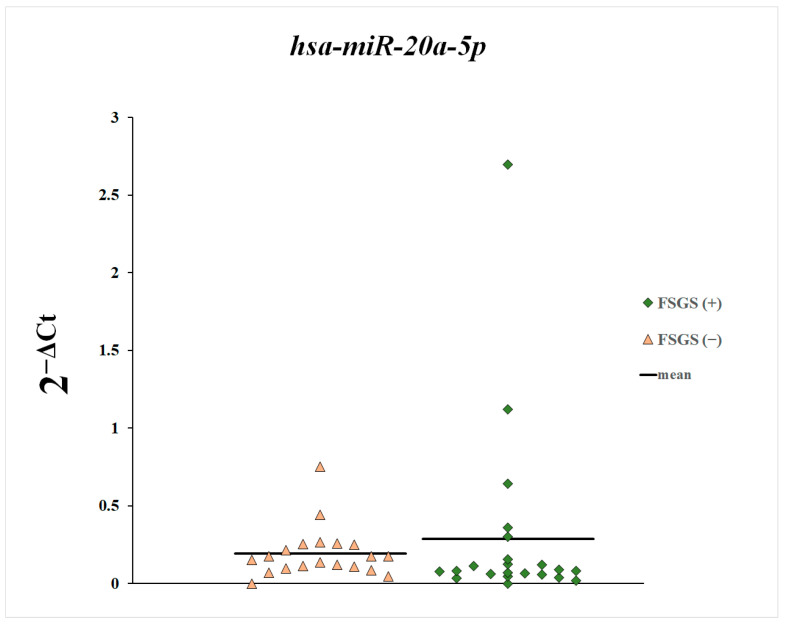
Plasma levels of miR-20a-5p in patients with and without diagnosed focal segmental glomerulosclerosis.

**Figure 5:**
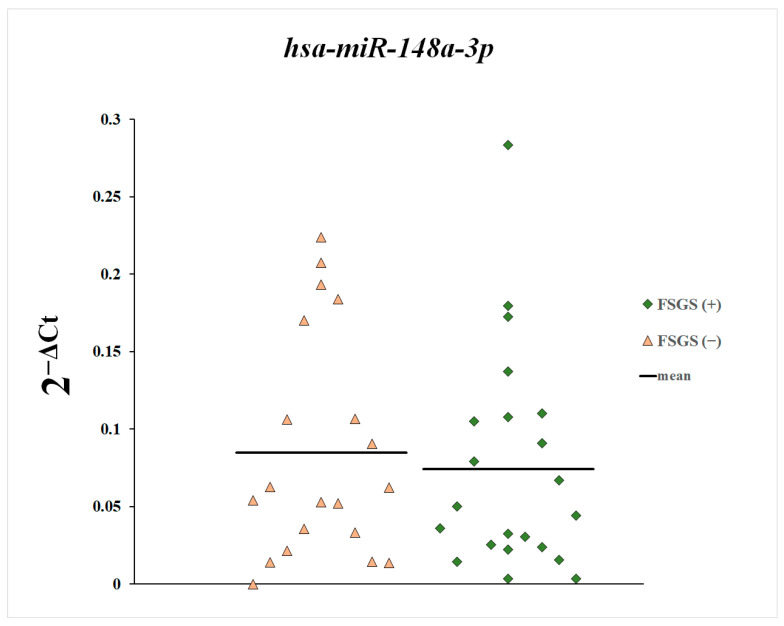
Plasma levels of miR-148a-3p in patients with and without diagnosed focal segmental glomerulosclerosis.

**Figure 6:**
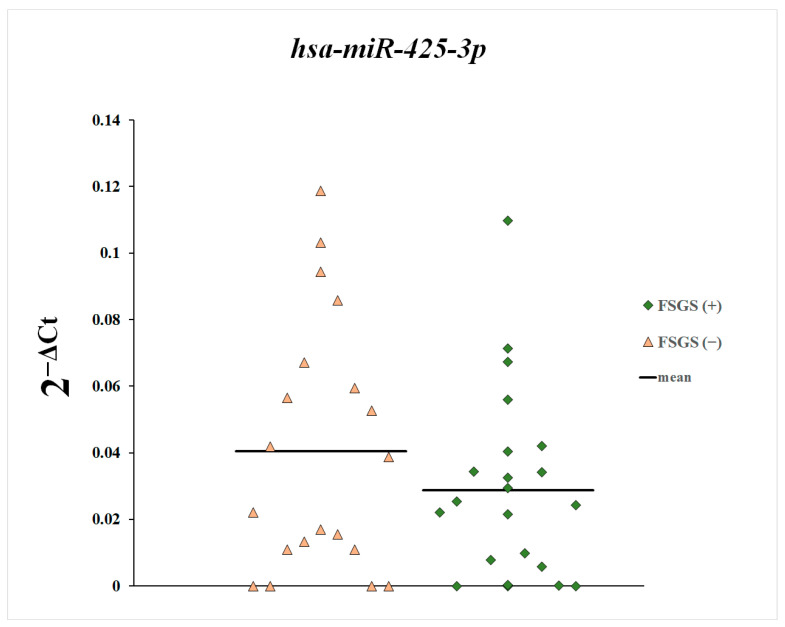
Plasma levels of miR-425-3p in patients with and without diagnosed focal segmental glomerulosclerosis.

Next, we compared the plasma levels of miR-148a-3p, miR-425-3p, and miR-20a-5p between patients with and without diagnosed focal segmental glomerulosclerosis (Figure 4, Figure 5 and Figure 6). These differences were statistically non-significant.

**Figure 7:**
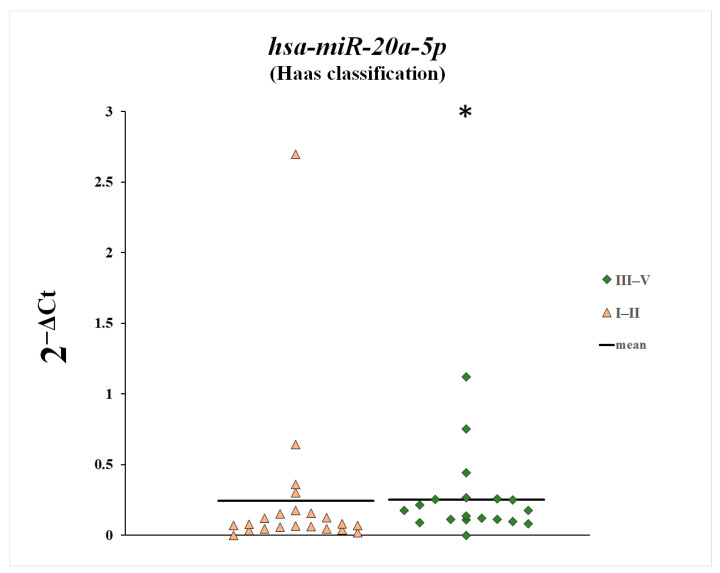
Plasma levels of miR-20a-5p in patients with histopathological changes classified as Stage 1 and 2 according to the Haas classification and in patients with Stages 3, 4, and 5, * *p* = 0.03, Mann–Whitney U test.

**Figure 8:**
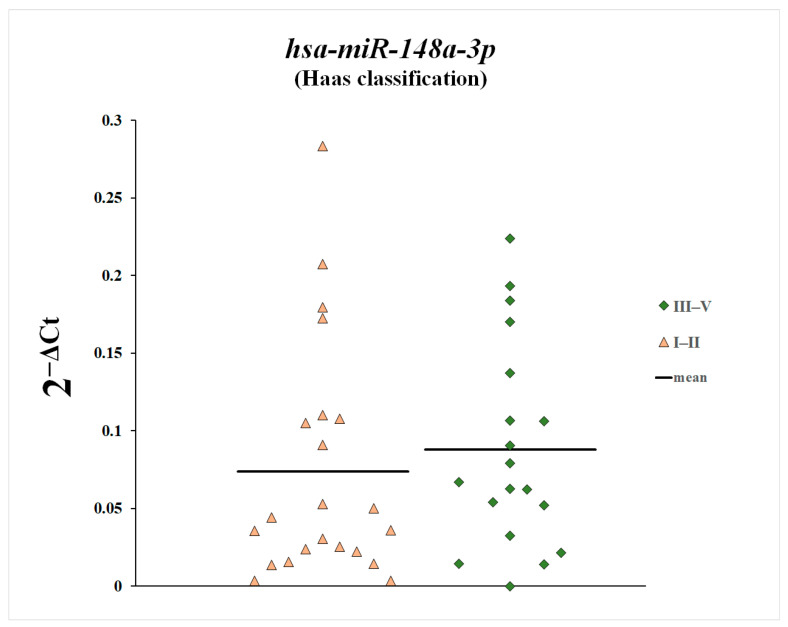
Plasma levels of miR-148a-3p in patients with histopathological changes classified as Stage 1 and 2 according to the Haas classification and in patients with Stages 3, 4, and 5.

**Figure 9:**
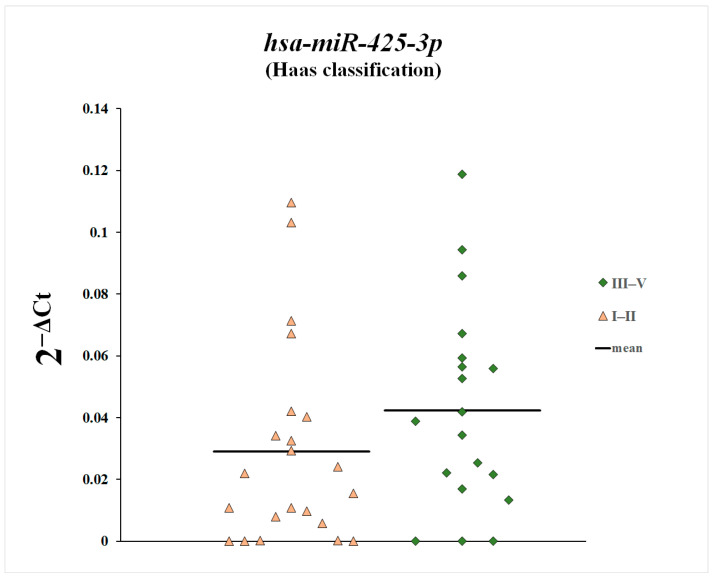
Plasma levels of miR-425-3p in patients with histopathological changes classified as Stage 1 and 2 according to the Haas classification and in patients with Stages 3, 4, and 5.

We also compared plasma levels of miR-148a-3p, miR-425-3p, and miR-20a-5p between patients with histopathological changes classified as Stage 1 and 2 according to the Haas classification and patients with Stages 3, 4, and 5 (Figure 7, Figure 8 and Figure 9). Plasma levels of miR-20a-5p were statistically significantly increased in patients with histopathological changes classified as Stages 3, 4, and 5 according to the Haas classification compared with patients with histopathological changes classified as Stages 1 and 2.

Next, we assessed the correlations between plasma levels of miR-148a-3p, miR-425-3p, and miR-20a-5p and clinical parameters in patients with IgA nephropathy. Plasma levels of miR-148a-3p and miR-425-3p in patients with IgA nephropathy correlated statistically significantly negatively with GFR values (Table 2, Table 3 and Table 4).

## 4. Discussion

IgA nephropathy (IgAN) is the most common primary proliferative glomerulonephritis, the pathogenesis of which is mediated by the deposition of immune complexes in the glomeruli. The resulting immune complexes cause inflammation, leading to changes in the glomerulus and subsequent glomerulosclerosis. The exact pathogenesis of this disease is not yet fully understood, but it is thought that a history of bacterial infection may be one of the causes leading to the formation of immune complexes [4].

The immune complexes formed are deposited in the mesangium, causing its proliferation and increasing the synthesis of pro-inflammatory cytokines, which leads to an increase in inflammation. Some microRNAs have been identified as playing an important role in the pathogenesis of the inflammatory response. Although they are not protein-coding, they influence the expression of many genes at the post-transcriptional level [13,14]. MicroRNAs act by binding to response elements in the 3′-untranslated region (3′-UTR), inhibiting mRNA translation. MicroRNAs are involved in modulating the expression of many genes and are therefore responsible for several processes that may influence the development of many different diseases [15]. The action of miRNAs is multidirectional, as they can affect various signaling pathways, metabolic processes, and cell apoptosis in multiple ways. They can be regulators of many processes in the human organism, as well as their markers [36].

To date, miRNAs have been used to monitor the clinical course of various diseases and offer the possibility of influencing their course, being a promising therapeutic target [36]. The impact of miRNAs on human body processes varies widely and depends on the type of tissue and cells involved and the influence of many other factors. It has been shown that disruption of miRNAs may be involved in developing many different diseases, including glomerulonephritis [37,38]. MicroRNAs may influence glomerular inflammation, mesangial proliferation, and infiltration. They may also be an early marker for the development of glomerular lesions. To date, the role of miRNAs has been confirmed by several studies in animal and cellular models. Still, there are only a few clinical studies assessing the role of miRNAs in the pathogenesis and clinical course of glomerulonephritis [39].

In this study, we examined plasma levels of miR-148a-3p, miR-425-3p, and miR-20a-5p in patients with IgA nephropathy. We have shown increased levels of miR-148a-3p and miR-425-3p in patients with IgA nephropathy in comparison with the control group. Moreover, plasma levels of miR-148a-3p and miR-425-3p in patients with IgA nephropathy correlated negatively with GFR values. We also found that plasma levels of miR-20a-5p were statistically significantly elevated in patients with histopathological lesions classified as Stages 3, 4, and 5 according to the Haas classification, compared with patients with histopathological lesions classified as Stages 1 and 2.

Increased plasma levels of miR-148a-3p and miR-425-3p in patients with IgA nephropathy and their negative correlation with GFR may suggest that plasma levels of these microRNAs increase with loss of glomerular function and the severity of the disease process. We also found elevated levels of miR-148a-3p and miR-425-3p in patients with histopathological lesions classified as Stages 3, 4, and 5 according to the Haas classification compared with patients with histopathological lesions classified as Stages 1 and 2, although these differences did not reach statistical significance. These results may suggest that miR-148a-3p and miR-425-3p may be markers of glomerular function and the glomerular filtration rate and that their plasma levels increase with the progression of the disease process and loss of glomerular function.

The detection of elevated plasma levels of miR-148a-3p and miR-425-3p in patients with IgA nephropathy and their negative correlation with GFR is not conclusive as to whether increased levels of these microRNAs and their negative correlation with GFR values are markers of IgA nephropathy or of chronic kidney disease and loss of renal function. In order to unequivocally confirm the role of these microRNAs in the pathogenesis of IgA nephropathy, it would be necessary to determine the expression of these microRNAs in the glomerular cells of patients with different stages of this disease and to compare these findings with patients with other forms of glomerulonephritis.

With regard to miR-20a-5p, we found that its plasma concentration was elevated compared with the control group, but the differences were not statistically significant. On the other hand, plasma miR-20a-5p levels were statistically significantly elevated in patients with histopathological changes classified as Stages 3, 4, and 5 according to the Haas classification compared with patients with histopathological changes classified as Stages 1 and 2. This indicates that plasma levels of miR-20a-5p increase with disease progression and glomerular lesion enhancement.

To date, many studies have been published on the role of miR-148a-3p, miR-425-3p, and miR-20a-5p in the pathogenesis of various diseases, such as cancer, cardiovascular disease, inflammatory diseases, and kidney disease [23,40,41,42,43,44,45]. So far, only one study performed in a Chinese population has confirmed the possible involvement of miR-148a-3p, miR-425-3p, and miR-20a-5p in the pathogenesis of IgA nephropathy [18]. However, due to the involvement of these microRNAs in various processes and signaling pathways that may contribute to the pathogenesis of IgA nephropathy, it seems appropriate to assess the role of these microRNAs in the pathogenesis of this disease.

Studies in a lupus nephritis model showed that miR-148a-3p expression was significantly increased in the glomeruli and serum of mice with lupus nephritis. At the same time, there was a positive correlation between miR-148a-3p and nuclear antigen expression of proliferating cells in the glomeruli [46]. It was shown that miR-148a-3p increases cell proliferation, while an inhibitor of miR-148a-3p decreases cell proliferation via the Akt/cyclin D1 pathway. Blocking miR-148a-3p was shown to inhibit glomerular fibrosis and improve renal function in mice with lupus nephritis by inhibiting the PTEN gene, indicating that miR-148a-3p increases cell proliferation and glomerular fibrosis in mice with lupus nephritis.

Li et al. examined the role of microRNAs in the pathogenesis of renal fibrosis. These authors indicated that miR-148a-3p may enhance renal fibrosis by increasing the expression of pro-inflammatory mediators [47]. Animals with renal fibrosis showed decreased SOD activity and increased expression of TGF-β1, TNF, VEGF, Smad3, and Smad7, which correlated with increased expression of miR-148a-3p.

Wu et al. examined microRNA levels in the plasma of patients with IgA nephropathy from a Chinese population. These authors found increased miR-148a-3p, miR-425-3p, and miR-20a-5p expression in the plasma of patients with IgA nephropathy, suggesting that miR-148a-3p, miR-425-3p, and miR-20a-5p may be the biomarkers of this disease [18].

Apart from the abovementioned study, miR-425-3p has not yet been investigated in glomerulonephritis and other kidney diseases. The involvement of miR-425-3p in the pathogenesis of some cancers and their therapies and other diseases has been confirmed [41,47,48]. It has been shown that miR-425-3p influences the TGF-β1 pathways [49], which may be involved in pathogenesis of IgA nephropathy [50,51].

Previous studies have found the association of miR-20a-5p with cancers, cardiovascular diseases, diseases of the respiratory tract, and some kidney diseases [52,53]. Koide et al. indicated the association of miR-20a-5p with abdominal aortic calcification in patients with chronic kidney disease [54]. These authors showed that miR-20a-5p affects VEGFA signaling pathways, which are involved in osteoblast differentiation and angiogenesis, as well as the development of chronic kidney disease. Shi et al. found increased expression of miR-20a-5p in renal transplant patients with DGF (delayed graft function). The authors demonstrated that miR-20a-5p inhibits the activity of long-chain acyl-CoA synthetase 4 (ACSL4), thereby inhibiting ACSL4-dependent ferroptosis [21].

The authors of another study detected an association of miR-20a-5p with ischemia-reperfusion injury. Reduced expression of miR-20a-5p was detected in the kidneys after ischemia-reperfusion injury. It has been shown that miR-20a-5p regulates ATG16L1 activity [55]. Increased expression of miR-20a-5p resulted in decreased LC3-II and ATG16L1 during hypoxia. These authors suggest that signaling pathways associated with miR-20a-5p and ATG16L1 may influence the mechanisms associated with ischemic renal injury during reperfusion. Deng et al. demonstrated the association of miR-20a-5p with acute kidney injury (AKI), which is the most common complication of sepsis. Expression of miR-20a-5p was significantly reduced in a mouse model of sepsis, and miR-20a-5p was shown to regulate NLRP3 activity while blocking miR-20a-5p-enhanced LPS-induced cellular pyroptosis. The authors suggested an important role for the miR-20a-5p/NLRP3 pathway in the development of sepsis in patients with acute kidney injury [55].

Chen et al. found increased miR-20a-5p expression in patients with chronic allograft dysfunction after renal transplantation [56]. These authors identified the effect of miR-20a-5p on the TGF-β signaling pathways that are associated with the development of tissue fibrosis. Muendlein et al. investigated the association of miR-20a-5p with renal function based on the estimated glomerular filtration rate (eGFR) in patients undergoing coronary angiography. It was found that miR-20a-5p influences eGFR values and is significantly associated with renal function in patients undergoing coronary angiography [57].

Although there are few clinical studies to date, previous studies in animal models and cell cultures demonstrate the possible involvement of these microRNAs in pathologies associated with the development of glomerulonephritis, including IgA nephropathy. Our results show increased expression of miR-148a-3p and miR-425-3p in the plasma, which seems to confirm the results obtained in the Caucasian population and in the Chinese population [18].

The demonstrated negative correlation with eGFR values and the increase in plasma levels of these microRNAs in patients with advanced histopathological changes suggest that the synthesis of these microRNAs increases with the development of the disease state. Although no differences in plasma levels of miR-20a-5p were found between the patient and control group, an increase in plasma levels of this microRNA was demonstrated with increasing histopathological changes in the kidney and progression of the disease.

However, our study has several limitations. This study does not resolve whether the elevated microRNA levels in patients with IgA nephropathy and their negative correlation with GFR values are a result of IgA nephropathy or a consequence of chronic kidney disease. Another limitation is the lack of an assessment of the change in the levels of each miRNA over time and the lack of determination of these microRNAs in glomerular cells. However, we hope that our results will prompt further research into the role of microRNAs in the pathogenesis of IgA nephropathy.

It is necessary to understand the mechanisms leading to increased synthesis of these microRNAs in IgA nephropathy and to validate, in large clinical studies, the usefulness of these microRNAs in possibly monitoring the disease process.

## 5. Conclusions

The results of our study indicated that in patients with IgA nephropathy, the increased plasma levels of miR-148a-3p and miR-425-3p correlated negatively with eGFR values. Plasma levels of miR-20a-5p were statistically significantly increased in patients with histopathological changes classified as Stages 3, 4, and 5 according to the Haas classification in comparison with patients with histopathological changes classified as Stages 1 and 2. However, confirmation of the involvement of these microRNAs in the pathogenesis of IgA nephropathy requires further study.

## Figures and Tables

**Table 1:** List of TaqMan™ Advanced miRNA assays used for qPCR analysis.

Assay Name	Assay ID
ath-miR159a	478411_mir
hsa-miR-191-5p	477952_mir
hsa-miR-20a-5p	478586_mir
hsa-miR-148a-3p	477814_mir
hsa-miR-425-3p	478093_mir

**Table 2:** Correlations between miR-148a-3p expression in the blood plasma and clinical parameters in patients with IgA nephropathy.

Parameters Correlated with Plasma Expression of miR-148a-3p	R_s_	*p*
Age [years]	0.21	0.16
WBC [G/L]	0.01	0.98
RBC [T/L]	0.04	0.85
HGB [mmol/L]	−0.01	0.97
HCT [L/L]	−0.01	0.96
PLT [G/L]	−0.02	0.93
Creatinine level [mg/dL]	0.20	0.20
Glomerular filtration rate [mL/min]	−0.30	0.04
Daily urinary protein loss [g/24 h]	−0.03	0.88

R_s_—Spearman’s rank correlation coefficient.

**Table 3:** Correlations between miR-425-3p expression in the blood plasma and clinical parameters in patients with IgA nephropathy.

Parameters Correlated with Plasma Expression of miR-425-3p	R_s_	*p*
Age [years]	0.23	0.13
WBC [G/L]	0.03	0.88
RBC [T/L]	−0.40	0.07
HGB [mmol/L]	−0.37	0.08
HCT [L/L]	−0.40	0.06
PLT [G/L]	0.08	0.74
Creatinine level [mg/dL]	0.25	0.10
Glomerular filtration rate [mL/min]	−0.36	0.01
Daily urinary protein loss [g/24 h]	0.06	0.74

R_s_—Spearman’s rank correlation coefficient.

**Table 4:** Correlations between miR-20a-5p expression in the blood plasma and clinical parameters in patients with IgA nephropathy.

Parameters Correlated with Plasma Expression of miR-20a-5p	R_s_	*p*
Age [years]	0.23	0.13
WBC [G/L]	−0.29	0.19
RBC [T/L]	−0.24	0.29
HGB [mmol/L]	−0.18	0.41
HCT [L/L]	−0.29	0.18
PLT [G/L]	−0.15	0.50
Creatinine level [mg/dL]	0.01	0.95
Glomerular filtration rate [mL/min]	−0.14	0.37
Daily urinary protein loss [g/24 h]	−0.09	0.61

R_s_—Spearman’s rank correlation coefficient.

## Data Availability

The original contributions presented in the study are included in the article, further inquiries can be directed to the corresponding author.
